# Preventing School Dropouts Should Start in Preschool

**Published:** 2008-03-15

**Authors:** Kevin Fiscella

**Affiliations:** Family Medicine, Community & Preventive Medicine and Oncology, University of Rochester, Rochester NY

## To the Editor:

In "Reframing School Dropout as a Public Health Issue" (1), Freudenberg and Ruglis make a succinct, yet compelling, case. Arguably, reducing racial, ethnic, and socioeconomic disparities in educational achievement (including disparities related to graduating from high school by age 18 years) is key to reducing not only disparities in adult incarceration and socioeconomic position but also to reducing disparities in adult health.

It is time to include school dropout rates (by race, ethnicity, and parental education and poverty level) among the national health objectives for 2010 (2). On the education side of the public health-education divide, it is equally important to acknowledge the contribution of child health (3) to the success of No Child Left Behind (4) in eliminating disparities in academic achievement (5).

Uniform national measures of school readiness (stratified by race, ethnicity, and parental education and poverty levels) should also be included among current national health objectives and tracked through No Child Left Behind. Such measures would underscore the relevance of children's emotional and cognitive health to academic achievement (and the relevance of academic achievement to adult health). It would also provide schools with baselines for assessing students' academic growth, beginning in kindergarten. Most importantly, it would offer a national benchmark for assessing progress toward the national goal of ensuring that every child starts elementary school with the necessary skills to succeed and eventually graduate from high school by age 18.

To the list of recommendations cited by the authors (1) for reducing school dropout rates, I strongly emphasize the need to add high-quality early-childhood interventions to reduce well-documented racial, ethnic, and socioeconomic disparities in school readiness (6,7). School dropouts, including those precipitated by adolescent pregnancy, often have their antecedents in inadequate readiness for school (8). Effective early childhood interventions exist (9); we just need the national will to fund their full implementation.

Early childhood interventions are critical to reducing school dropouts over the long term. These programs are highly cost effective (10) and may prove to be one of the most powerful tools for reducing upstream causes of disparities in adult health.

**Read the author's reply**
